# Reporting activities for the oxygen evolution reaction

**DOI:** 10.1038/s42004-023-01024-y

**Published:** 2023-10-14

**Authors:** Marcel Risch

**Affiliations:** Nachwuchsgruppe Gestaltung des Sauerstoffentwicklungsmechanismus, Helmholtz-Zentrum Berlin, Hahn-Meitner Platz 1, 14109 Berlin, Germany

**Keywords:** Electrocatalysis, Hydrogen fuel, Electrocatalysis, Electrocatalysis, Batteries

## Abstract

The oxygen evolution reaction (OER) is a key enabler of sustainable chemical energy storage. Here, the author assesses the current status of protocols for benchmarking the OER in materials- and device-centered investigations and makes suggestions for more comparable data.

## Significance of the oxygen evolution reaction

**Fig. 1:**
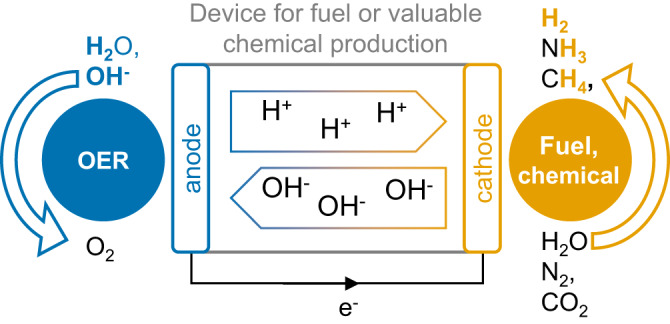
Versatile uses of the oxygen evolution reaction (OER) for the production of fuels and chemicals. The anodic OER provides the ions needed for the cathodic reduction of precursors such as water, nitrogen, or carbon dioxide to the desired fuel or chemical.

Sustainable, climate-friendly, alternatives to fossil resources are needed to meet the needs of the energy and chemical sectors. Precursor feeds of non-potable water^1^ and in some cases aqueous nitrogen or carbon dioxide could be electrochemically reacted to sustainably produce many key fuels and valuable chemicals from renewable sources used in devices such as electrolyzers or photoelectrochemical cells (Fig. 1). The water oxidation reaction (WOR) or oxygen evolution reaction (OER) at the anode takes a pivotal role in this approach as it provides the protonated ions for the reduction of precursors at the cathode to the desired fuel or chemical. Four electrons and ions need to be transferred to make O_2_ from 2H_2_O (or 4OH^-^ in alkaline media), at the cost of large overpotential at the fuel- or chemical-producing cathode. Additionally, the needed high potentials to drive the OER may degrade various electrode components^2^. Therefore, the identification of stable and active electrocatalysts for the OER has received considerable interest in the last decades. While stability and activity are both important and usually linked, this Comment focuses on reporting the activity of OER electrocatalysts in the context of the most mature field of water electrolysis and in particular the issue of comparability among reported activities in current benchmarking studies.

## Benchmarking protocols

**Fig. 2:**
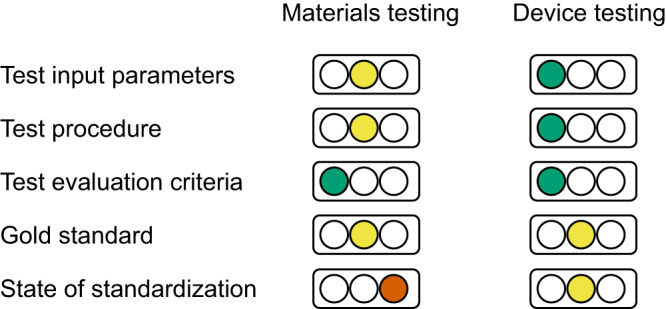
Current state of benchmarking protocols and standardization. Green (left) indicates sufficient definition, yellow (middle) indicates further work needed, and red (right) indicates a lack of the criterion.

Bligaard et al.^3^ define benchmarking in catalysis as a “community-based and (preferably) community-driven activity involving consensus-based decisions on how to make reproducible, fair, and relevant assessments […] between new and standard catalysts”. Building on the latter definition and previous implementation^4^, this author proposes that a complete benchmarking protocol should contain (1) a definition of all relevant test input parameters and environmental conditions; (2) a protocol of the test procedure, i.e., the sequence of measurements to be performed; (3) a concise definition of the test output parameters and their evaluation criteria; (4) a well-defined and readily accessible gold standard. The current state toward a complete protocol is depicted in Fig. 2 for materials-centered research and device-centered research on the OER.

Several protocols have been published for materials-centered investigations of the OER. The protocol reported by McCrory et al.^5^ is the most widely used, yet still only by an insignificant fraction of all OER publications. Other protocols were reported by Spanos et al.^6^, Burke-Stevens et al.^7^, Peugeot et al.^8^, and Creel et al.^9^, as well as recommendations by Wei et al.^10^ (Table 1). The published protocols and the majority of materials-centered OER studies use potential sweeps (cyclic or linear sweep voltammetry) for conditioning, surface area determination (note the pitfalls^11^), and activity determination. The protocols include either additional current and/or potential steps for activity determination or alternative current and/or potential steps for this purpose.

**Table 1:** Overview of benchmarking protocols.

Protocol	Step 1	Step 2	Step 3	Step 4	Step 5	Step 6	Step 8
McCrory 2013^5^	CV DL, 100 mV range centered around OCP, no rotation	EIS, no rotation	CV (1.23 to 1.82 V vs. RHE) at 10 mV/s, 1600 rpm, O2	Potential steps (1.23 to 1.82 V vs. RHE, 30 s), 1600 rpm, O2	Current steps (0.1 to 20 mA/cm^2^, 30 s), 1600 rpm, O2	2 h at 10 mA/cm^2^, 1600 rpm, O2	n/a
Spanos et al. 2017 A^6^	OCP, LSV to start position of conditioning	Condition the catalyst	EIS at OCP, LSV from OCP to 1.2 V vs. RHE, 5 mV/s	LSV 1.2 to 1.7 V vs. RHE, 5 mV/s	Passing 1 C charge by CP or CA, LSV OCP to 1.8 V vs. RHE, 5 mV/s	2 h at 10 mA/cm^2^ with ICP-OES analysis, flow rate of 0.86 mL/min	Repeat steps 3–5
Spanos et al. 2017 B^6^	OCP, LSV to start position of conditioning	Condition the catalyst	EIS at OCP, LSV from OCP to 1.2 V vs. RHE, 5 mV/s	LSV 1.2 to 1.7 V vs. RHE, 5 mV/s	Passing 1 C charge by CP or CA, LSV OCP to 1.8 V vs. RHE, 5 mV/s	2 h at 1.8 V vs. RHE with ICP-OES analysis, flow rate of 0.86 mL/min	Repeat steps 3–5
Burke-Stevens et al. 2017^7^	CV (0.93 to 1.68 V vs. RHE), 10 mV/s	EIS at 1.53 V vs. RHE	current steps from 0.01 mA/cm^2^ to 10 mA/2 for 3 min	current steps from 10 mA/2 to 0.01 mA/cm^2^ for 3 min	Repeat steps 1–2	CA at 1.53 for 1 h	n/a
Malkov 2018 A PEMWE^4^	0.1 A/cm^2^, 5 min	linear current sweep from 1 mA/cm^2^ to 2000 mA/cm^2^ at 0.080 A/cm^2^ per min, 2 V cutoff	n/a	n/a	n/a	n/a	n/a
Malkov 2018 A AWE + AEMWE^4^	0.1 A/cm^2^, 5 min	linear current sweep from 0.2 mA/cm^2^ to 400 mA/cm^2^ at 0.016 A/cm^2^ per min, 2 V cutoff	n/a	n/a	n/a	n/a	n/a
Malkov 2018 B PEMWE^4^	0.1 A/cm^2^, 5 min	current steps from 1 mA/cm^2^ to 2000 mA/cm^2^, with 30 s dwell and 30 s acquisition per step, 2 V cutoff	n/a	n/a	n/a	n/a	n/a
Malkov 2018 B AWE + AEMWE^4^	0.1 A/cm^2^, 5 min	current steps from 0.2 mA/cm^2^ to 400 mA/cm^2^, with 30 s dwell and 30 s acquisition per step, 2 V cutoff	n/a	n/a	n/a	n/a	n/a
Bender et al. 2019^12^	0.2 A/cm^2^ for 30 min, 1 A/cm^2^ for 30 min, 1.7 V until variation less than 1% per h	0.0 to 0.1 A/cm^2^ in 0.02 A/cm^2^ steps and 0.2 A/cm^2^ steps above until 2 V are reached,5 min, optional: EIS at all steps	Reverse of step 2	Optional: OCV	n/a	n/a	n/a
Wei et al. 2019 A^10^	O2 bubbling for 10–30 min	CV (1.0 to 1.7 V vs. RHE) at 10 mV/s, 1600 rpm, O2	n/a	n/a	n/a	n/a	n/a
Wei et al. 2019 B^10^	O2 bubbling for 10–30 min	CA with at least 5 potentials, for which *j* < 2.5 mA/cm^2^	n/a	n/a	n/a	n/a	n/a
Peugeot et al. 2021^8^	LSV at 10 mV/s until a steady response	0, 5, 10, 25, 50, 100 mA/cm^2^ for 5 min, extension of duration if potential was not stable	50 mA/cm^2^ for 30 min	n/a	n/a	n/a	n/a
Creel 2022^9^	Condition the catalyst	LSV (1.4 V to 2.2 V vs. RHE) at 10 mV/s, inert gas	EIS at OCP, OCP-50 mV, OCP + 50 mV	CV DL (step 1 of McCrory 2013)	0 mA for 3 s, 20 mA for 1 h, 0 A for 1 s	n/a	n/a
Risch 2023, this work	Condition the catalyst according to (sub) community	0 to 50 mA/cm^2^ in 5 mA/cm^2^ steps with at least 1.55 V (vs. RHE for 3-electrode or counter for 2-electrode), 100 mA/cm^2^ and 100 mA/cm^2^ steps above until 2 V (vs. RHE for 3-electrode or counter for 2-electrode) are reached, 30 s dwell, 30 s acquisition, optional: EIS at all steps	reverse of step 2	Optional: EIS or surface area measurement	Optional: determine Faradaic efficiency if not included in step 2	n/a	n/a

A, B, PEMWE, AWE+AEMWE denote variants of the protocol in the same report.

For device-centered investigations of the OER testing, Malkow et al.^4^ published protocols for testing low-temperature water electrolyzers that employ galvanostatic sweeps or a list of current density setpoints (Table 1). A similar protocol was used in a round-robin study^12^. Higher current densities are included but the range of current densities overlaps with that of the materials-centered investigations. Additional definitions and experimental parameters are published in a series of reports by Tsotridis and Pilenga^13,14^ that are partly based on definitions of the International Electrochemical Commission (IEC) such as standard IEC TS 60050-485:2020.

## Assessment of the protocols

The test input parameters are defined in protocols for both materials and device testing with sufficient detail for reproduction. More environmental conditions are controlled for device testing. It will improve materials testing to mandate control of environmental conditions such as temperature, which is readily available through jacketed electrochemical cells. In the materials-centered protocols, it is not specified how the electrochemical data is sampled, i.e., whether the current/potential reading occurs at the end of the sampling interval or by integration. This can drastically affect the contribution of capacitive currents in sweep measurement or short pulses on (desirable) high surface area materials, which would lead to an overestimation of activity metric based on electrochemical current.

The procedures vary for materials-centered testing, where potential sweeps as well as potential and current holds are performed in various combinations. The author expects that this is the main issue that reduces comparability among the protocols because different surfaces can be formed by sweep and potential/current holds^15^ and because the range of potential sweeps may affect the measured currents^16^. Wei et al.^10^ and Malkow et al.^4^ recommend either sweeps or holds. This author recommends several current holds, i.e., a Tafel plot (note the pitfalls^17^), with increasing current density until 2 V is reached and sufficient duration to ensure a steady state of the double layer and electrocatalyst microstructure (electronic structure, phase, and morphology). Using current holds for both materials- and device-centered investigations to determine the activity metric(s) can be a small step towards crossing the gap between fundamental and applied research.

Output parameters and evaluation criteria are clearly defined in the previous reports for both materials- and device-centered investigations. Common activity metrics are various (over)potentials at fixed current (density) or current (densities) at fixed (over)potential where the current is normalized by a property of the used electrocatalyst material (e.g., electrocatalyst mass or surface area) or a property of the electrode (e.g., electrode area). In addition to electrochemical data, this author urges to also report a measure of the evolved oxygen or the Faradaic efficiency^5,6^. The focus on specific activity metrics and reporting recommendations differ in details but several protocols include Tafel plots as recommended above, from which a desired metric could be calculated, most readily if the electrochemical data was published openly and FAIR (findable, accessible, interoperable, reusable)^18^ in a data repository.

## Importance of standards

To date, gold standard materials for the OER are Ni-Fe oxides in alkaline and RuO_2_ as well as IrO_2_ in acid. Unfortunately, the outcome of their test evaluation criteria depends strongly on details of synthesis, possible non-electrochemical post-treatment steps as well as electrochemical conditioning steps. For Ni-Fe oxide, a simple synthesis has been reported^7^. Powders of these oxides and membrane electrode assemblies (MEA) based on iridium-ruthenium oxide are also available commercially. Issues with preparation aside, there is no standard electrocatalyst or electrode consistently used in all reported protocols. Ideally, the field would need a benchmark akin to the international prototype of the kilogram and its exact copies, which would enable to comparison of the reported protocols. In the field of photovoltaics, testing centers such as the European Solar Test Installation (ETSI) have been established where one sends samples for standardized tests, thus eliminating the considerable variation observed in round-robin tests (on electrolyzers)^12^. A clearly defined gold standard and standardized testing, especially in specialized facilities, would significantly improve the reliability of reported OER activity metrics to benchmark electrocatalysts and electrodes, identify structure-property relationships, and harness big data analysis in electrocatalysis.

In summary, the state of standardization of materials-centered investigations of the OER is less advanced as compared to device-centered investigations, yet there are no international formalized standards such as the ones that exist in corrosion science, e.g., ASTM G150-18 or DIN EN ISO 17864:2008-07, for either community. For materials testing, there are additionally no harmonized protocols or no round-robin studies on gold standards. Furthermore, most reports of highly active materials unfortunately do not follow any of the reported protocols to obtain their activity metric(s).

## Outlook

As pointed out by Bligaard^3^ benchmarking must be a community-driven effort. This raises the question of which are the relevant communities and should we thrive to identify a universal protocol? This author believes that the conditioning part of the protocol should be defined by sub-communities, e.g., catalyst ink investigations, epitaxial thin films, alkaline electrolyzers, etc. As recommended above, a current step protocol could better connect Tafel plots in materials- and device-centered OER investigations. Additional measurements could be performed after the Tafel plot or on separate samples, e.g., measurements of the Faradaic efficiency^5^. These recommendations (Table 1) should be seen as a seed for the needed discussion in the community rather than competition with previous protocols. Implementing a harmonized base protocol and gold standard would be comparably little effort with large gain for the community toward truly benchmarking the OER being important in many contexts beyond water electrolysis (Fig. 1).
